# Potential vestibular pathway impairment in children with recurrent vertigo: An investigation through air-conducted sound and galvanic vestibular stimulation-triggered vestibular evoked myogenic potentials

**DOI:** 10.3389/fneur.2022.997205

**Published:** 2022-10-10

**Authors:** Xiayu Sun, Dekun Gao, Jiali Shen, Qi Zhu, Lu Wang, Xiaobao Ma, Wei Wang, Xiangping Chen, Qing Zhang, Yulian Jin, Jianyong Chen, Jun Yang

**Affiliations:** ^1^Department of Otorhinolaryngology-Head and Neck Surgery, Xinhua Hospital, Shanghai Jiaotong University School of Medicine, Shanghai, China; ^2^Shanghai Jiaotong University School of Medicine Ear Institute, Shanghai, China; ^3^Shanghai Key Laboratory of Translational Medicine on Ear and Nose Diseases, Shanghai, China; ^4^Department of Otorhinolaryngology-Head & Neck Surgery, Yuyao People's Hospital, Yuyao, China

**Keywords:** recurrent vertigo of childhood, vestibular evoked myogenic potential, elicit rate, latency, saccule, utricle, vestibular pathway

## Abstract

**Objective:**

This study aims to investigate the potential vestibular pathway impairment through vestibular evoked myogenic potentials (VEMPs) and to explore the pathophysiological significance of these instrument-based findings in children with recurrent vertigo.

**Materials and methods:**

The clinical data of 21 children (mean age 4.67 ± 1.39 years) diagnosed as RVC who met the inclusion criteria of the Bárány Society and 29 healthy children (mean age 4.83 ± 1.34 years) enrolled as the control group from February 2021 to December 2021 were collected and analyzed retrospectively. All the subjects underwent both cervical VEMP (cVEMP) and ocular VEMP (oVEMP) triggered by air-conducted sound (ACS) and galvanic vestibular stimulation (GVS), respectively. The elicit rate, latency, and amplitude asymmetry ratio (AAR) of ACS-cVEMP, ACS-oVEMP, GVS-cVEMP, and GVS-oVEMP were analyzed.

**Results:**

(1) The elicit rates of ACS-cVEMP and ACS-oVEMP were similar in the two groups (*P* > 0.05), as well as GVS-cVEMP and GVS-oVEMP (*P* > 0.05). (2) P1 and N1 latencies of ACS-cVEMP and GVS-cVEMP in the RVC group were longer than those in the control group (*P* < 0.05). (3) The N1 latency of ACS-oVEMP in the RVC group was shorter than that in the control group (*P* < 0.05), while there was no significant difference in the P1 latency of ACS-oVEMP (*P* > 0.05). The N1 and P1 latencies of GVS-oVEMP were not significantly different (*P* > 0.05). (4) There was no statistical difference in the AAR of ACS-cVEMP and GVS-cVEMP. Although there was an increased AAR of ACS-oVEMP in the RVC group (*P* < 0.05), the AAR was within the normal range. However, no statistical difference was found in the AAR of GVS-oVEMP in the two groups (*P* > 0.05).

**Conclusion:**

The latencies of ACS-cVEMP and GVS-cVEMP in children with recurrent vertigo were significantly prolonged compared with those in healthy children, and there was no difference in elicit rates of ACS-cVEMP and GVS-cVEMP, suggesting that there might be potential impairment in the inferior vestibular nerve and the subsequent nerve conduction pathway in RVC.

## Introduction

The spectrum of vertigo diseases in children and adults is different. In children, the most common diseases that cause vertigo are vestibular migraine (VM) and benign paroxysmal vertigo of childhood (BPVC) (1, 2). BPVC, first reported by Basser in 1964 (3), is characterized by recurrent spontaneous attacks of vertigo, which may be associated with vomiting, pallor, fearfulness, postural imbalance, ataxia, and/or nystagmus in otherwise healthy children (4). Children with BPVC present with recurrent episodes of dizziness or vertigo, without accompanied by visual aura, photophobia, phonophobia, and ear symptoms such as tinnitus, aural fullness, hearing loss, or neurological symptoms such as change in consciousness.

The Committee of Vestibular Disorders of the Bárány Society and the International Headache Society released the latest diagnostic criteria of vertigo disorders in children in 2021. Since then, the diagnostic nomenclature BPVC has been replaced by “recurrent vertigo of childhood (RVC)”. The diagnostic criteria of RVC include (1) at least three episodes with vestibular symptoms of moderate or severe intensity, lasting between 1 min and 72 h; (2) none of the criteria for VM, with no history of migraine with or without aura, photophobia, and phonophobia; (3) age <18 years; and (4) not better accounted for by another headache disorder, vestibular disorder, or other conditions (4).

At present, the pathogenesis of RVC remains unclear. Some studies suggested that children with RVC have abnormal vestibular evoked myogenic potentials (VEMPs) (5–7), mainly reflected as the failure of elicitation and latency delay, indicating that there is a potential impairment in the otolith and vestibular nerve conduction pathway. VEMPs can be induced by air-conducted sound (ACS), bone-conducted vibration (BCV), and galvanic vestibular stimulation (GVS). Both ACS-VEMPs and BCV-VEMPs depend on the intactness of the otolith, while GVS directly stimulates the vestibular verve endings to elicit VEMPs. Therefore, those findings induced by different VEMPs can be compared to locate the lesion of the vestibular pathway in intra-labyrinthine or retro-labyrinthine (8). More often, GVS-VEMPs can be recorded in adults; however, there is no research on GVS-VEMPs in children (9, 10). Our study intends to investigate the potential vestibular pathway impairment through VEMPs and to explore the pathophysiological significance of these instrument-based findings in children with RVC.

## Materials and methods

### Design of the study

This study was conducted in the Department of Otorhinolaryngology-Head & Neck Surgery of Xinhua Hospital affiliated to Shanghai Jiaotong University School of Medicine, which was designed and conducted following the ethical standards of the Helsinki Declaration. It was completed from February 2021 to December 2021 after approval from the ethical committee of the institute (No. XHYY-2021-039).

The inclusion criteria for healthy children in the control group were as follows: (1) no history of dizziness, vertigo, or headache; (2) no history of brain disease and trauma; (3) no history of ear diseases; (4) pure tone average (500–2,000 Hz) in the normal range of 0–25dB HL; and (5) no cognitive impairment.

The inclusion criteria for children with RVC were (1) at least three episodes with vestibular symptoms of moderate or severe intensity, lasting between 1 min and 72 h; (2) no history of migraine with or without aura, photophobia, and phonophobia; (3) age <18 years; and (4) not better accounted for by another headache disorder, vestibular disorder, or other condition.

The exclusion criteria for children with RVC were as follows: (1) a history of benign paroxysmal positional vertigo, vestibular neuritis, Meniere's disease, and other peripheral vestibular vertigo diseases; (2) a history of VM and headache; (3) a history of known neurological diseases; (4) a history of ear diseases; and (5) unable to cooperate to complete VEMP tests.

A detailed explanation of the procedures that they may undergo was given to the participants, and a signed informed consent form was obtained from the guardian of each participant. ACS-VEMPs and GVS-VEMPs were applied to both ears of each participant who met the inclusion criteria.

### Participants

A total of 29 healthy children aged 3–9 (4.83 ± 1.34) years and 21 children with RVC aged 3–9 (4.67 ± 1.39) years participated. In total, 15 boys and 14 girls were in the control group, while 12 boys and 9 girls were in the RVC group. The distribution of girls and boys in each group was equal. All participants were evaluated with ACS-cVEMP, ACS-oVEMP, GVS-cVEMP, and GVS-oVEMP tests.

### ACS-VEMPs

The ACS-VEMP tests were performed using Neuropack MEB-9404 C (NIHON KOHDEN, Japan). A 500-Hz tone burst was given as a stimulus to obtain a VEMP response, and a rate of 5.1/s and an intensity of 105 dB nHL (132 pe SPL) were presented to the ipsilateral ear by air conduction insert earphones. The rise/fall time was 1 ms, and the plateau was 2 ms.

The cVEMP test was performed in the participants in a sitting position. They were required to turn their heads away from the stimulated ear in order to elicit an appropriate and replicable contraction level of the sternocleidomastoid muscle (SCM). Electrode placement: two record electrodes were placed symmetrically at the upper third of bilateral SCMs, the reference electrode was placed on the sternal end of the SCM, and the ground electrode was placed over the forehead. The oVEMP test was performed with participants in a sitting position with their heads kept straight. They were required to gaze at a maximal comfortable up-gaze position to elicit appropriate and replicable contraction level of the inferior oblique muscle (IOM). Electrodes placement: two record electrodes were placed symmetrically below the center of each lower eyelid, two reference electrodes were placed 2–3 cm inferior to the record electrodes and the ground electrode was placed over the forehead.

An electromyography (EMG) recording window also displayed the background muscle activity at the same time, which could reflect whether the muscle strength of SCM or IOM was maintained within the ideal range required for the test, which is usually above 50 mV for the SCM in children older 3 years (11).

The VEMP waveform have a positive and a negative peak, which are named P1 and N1, respectively. VEMP indices include elicit rate, P1 latency, N1 latency, P1-N1 amplitude, and amplitude asymmetry ratio (AAR). The P1 latency, N1 latency, and P1-N1 amplitude value were recorded on both ears of each participant. The AAR was calculated using the formula (AL -AS) / (AL + AS) × 100%, where AL is the larger P1-N1 amplitude value between two ears, while AS is the smaller one. In other words, AAR is the difference of bilateral amplitudes divided by the sum of bilateral amplitudes. The AAR value is between 0 and 1. The closer the value to 0, the better the symmetry of bilateral VEMPs. The closer the value to 1, the worse the symmetry of bilateral VEMPs, considering that there might be dysfunction of the unilateral otolith and vestibular nerve conduction pathway (12).

Our study set the upper limit standard of the normal AAR value of cVEMP to 33%, that is, when the P1-N1 amplitude of one ear is less than half that of the other ear, it is judged to be abnormal (13).

In the review on VEMPs written by Dlugaiczyk (14), it was mentioned that the “AAR value exceeding 40% indicates the asymmetry of bilateral utricle and the superior vestibular nerve conduction pathway”. Therefore, our study set the upper limit standard of the normal AAR value of oVEMP to 40%.

### GVS-VEMPs

The GVS-VEMP tests were performed using Neuropack MEB-9404 C (NIHON KOHDEN, Japan). The stimulus rate was 5 Hz, the stimulus duration was 1 ms, and the current level was 3 mA. For each trace, the number of stimuli was 100. EMG recordings were amplified for analysis. A 20- to 2,000-Hz bandpass filter and notch filter were applied on collected recordings. The analysis time window was 50 ms.

The GVS-VEMP tests were performed with the participants in a sitting position in two stages. In the first stage, when the SCM/IOM was not contracted, the first trace was obtained by sending the galvanic stimulus over the mastoid of the side being tested. In the second stage, when the SCM/IOM was contracted, the second trace was obtained by sending the galvanic stimulus. There were artifacts from the galvanic stimulus in both traces. Since these waveforms included very high artifacts, the subtraction method was used to eliminate artifacts. The first trace (without contraction of SCM/IOM) was subtracted from the second trace (with contraction of SCM/IOM), and finally, the GVS-VEMP waveforms were obtained (10).

Recording parameters were identical to those of ACS-VEMPs. For GVS-cVEMPs, two record electrodes were placed symmetrically at the middle of bilateral SCMs, a reference electrode was placed on the superior sternal fossa, a ground electrode was placed on the nasion, a negative stimulus electrode was placed on the mastoid, and a positive stimulus electrode was placed over the forehead. For GVS-oVEMPs, two record electrodes were placed symmetrically below the center of each lower eyelid, two reference electrodes were placed 2–3 cm inferior to the record electrodes, a ground electrode was placed on the nasion, a negative stimulus electrode was placed on the mastoid, and a positive stimulus electrode was placed over the forehead.

## Statistical analysis

The data were analyzed by IBM SPSS Statistics 26.0 (Chicago, IL, United States). The mean and standard deviation for latencies and amplitudes of VEMPs and the percentages of elicit rate and AAR were calculated. Parametric tests were used for all statistical analyses. Two independent sample *t*-tests were used for the comparison between the healthy children and children with RVC. The chi square test was used to compare the elicit rates. Statistical significance was set at *P* <0.05.

## Results

### General data of participants

This study was carried out with 29 healthy children (58 ears) and 21 children with RVC (42 ears). In the control group, a total of 15 boys and 14 girls participated in the study, while in the RVC group, 12 boys and nine girls were involved. No statistical significance was observed in the comparison of the gender between the two groups (χ^2^ = 0.144, *P* = 0.704). All the participants' ages ranged from 3 to 9 years, in which the age of the control group was 4.83 ± 1.34 years and the age of the RVC group was 4.67 ± 1.39 years. There was no significant difference in age between the two groups (*t* = 0.413, *P* = 0.682).

### Comparison of cVEMP elicit rates

**Figure 1:**
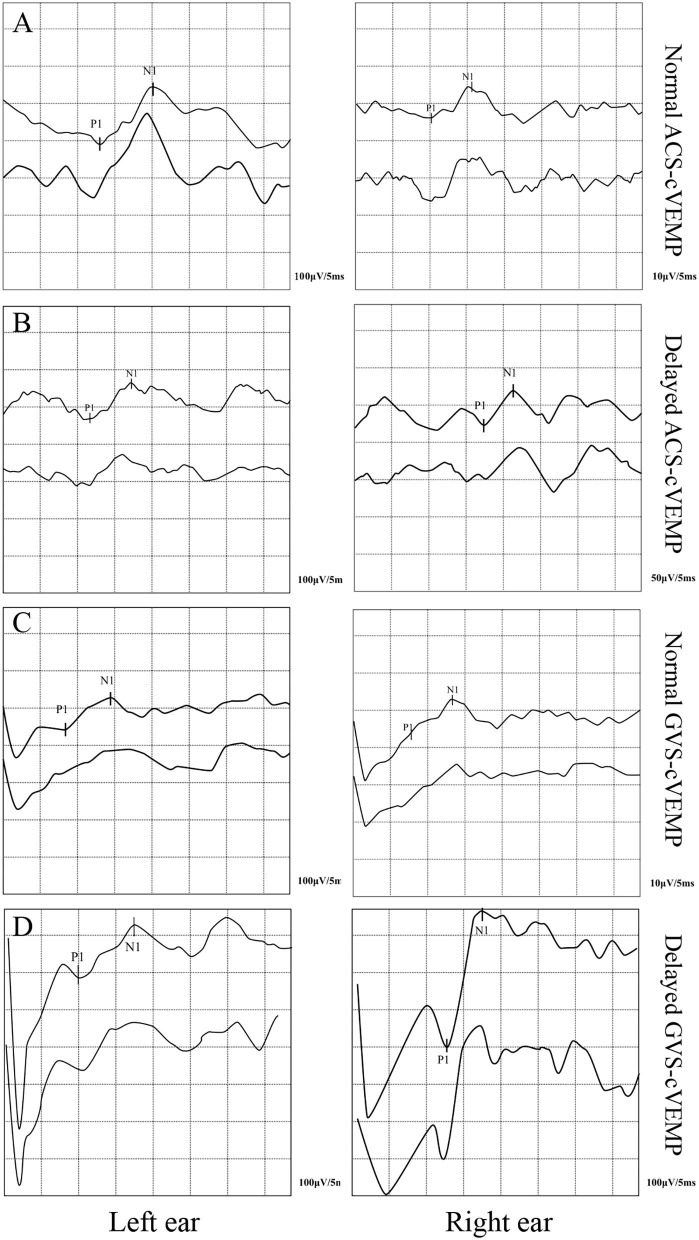
Typical double-trace records of cVEMPs. **(A)** Normal ACS-cVEMP. **(B)** Delayed ACS-cVEMP. **(C)** Normal GVS-cVEMP. **(D)** Delayed GVS-cVEMP.

The cVEMP elicit rates of the two groups are shown in Table 1. The ACS-cVEMP elicit rate was 98% in the RVC group and 97% in the control group, with no statistically significant difference between the two groups (χ^2^ = 0.095, *P* = 0.758). The GVS-cVEMP elicit rate was 98% in the RVC group and 93% in the control group, with no statistically significant difference between the two groups (χ^2^ = 1.046, *P* = 0.306). Typical results of ACS-cVEMP and GVS-cVEMP are shown in Figure 1.

**Table 1:** Comparison of cVEMP elicit rates between the RVC group and the control group.

**Group**	**ACS-cVEMP**				**GVS-cVEMP**			
	**Elicite (ears)**	**Not elicite (ears)**	**χ^2^**	**P**	**Elicite (ears)**	**Not elicite (ears)**	**χ^2^**	**P**
RVC	41	1	0.095	0.758	41	1	1.046	0.306
Control	56	2			54	4		

### Comparison of oVEMP elicit rates

**Figure 2:**
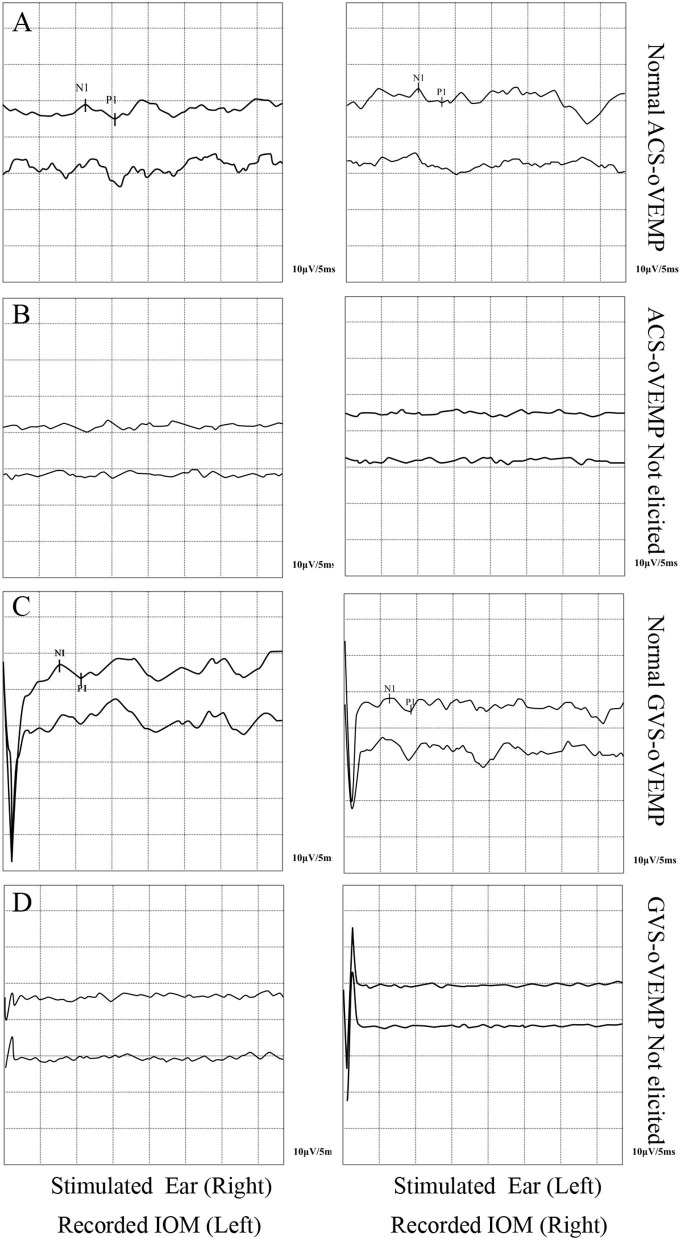
Typical double-trace records of oVEMPs. **(A)** Normal ACS-oVEMP. **(B)** Not elicited ACS-cVEMP. **(C)** Normal GVS-oVEMP. **(D)** Not elicited GVS-oVEMP.

The oVEMP elicit rates of the two groups are shown in Table 2. The ACS-oVEMP elicit rate was 90% in the RVC group and 83% in the control group, with no statistically significant difference between the two groups (χ^2^ = 1.205, *P* = 0.272). The GVS-cVEMP elicit rate was 95% in the RVC group and 88% in the control group, with no statistically significant difference between the two groups (χ^2^ = 1.588, *P* = 0.208). Typical results of ACS-oVEMP and GVS-oVEMP are shown in Figure 2.

**Table 2:** Comparison of oVEMP elicit rates between the RVC group and the control group.

**Group**	**ACS-oVEMP**				**GVS-oVEMP**			
	**Elicite (ears)**	**Not elicite (ears)**	**χ^2^**	**P**	**Elicite (ears)**	**Not elicite (ears)**	**χ^2^**	**P**
RVC	38	4	1.205	0.272	40	2	1.588	0.208
Control	48	10			51	7		

### Self-comparison of cVEMP and oVEMP elicit rates in the control group

The cVEMP and oVEMP elicit rates of the control group are shown in Table 3. The elicit rate of ACS-cVEMP was 97%, which was higher than the 83% value of ACS-oVEMP, with a statistical significance in the comparison (χ^2^ = 5.949, *P* = 0.015). The GVS-cVEMP and GVS-oVEMP elicit rates of the control group were 93 and 88%, respectively, with no statistical significance in the comparison (χ^2^ = 0.904, *P* = 0.342).

**Table 3:** Self-comparison of cVEMP and oVEMP elicit rates in the control group.

**VEMPs**	**ACS**				**GVS**			
	**Elicite (ears)**	**Not elicite (ears)**	**χ^2^**	**P**	**Elicite (ears)**	**Not elicite (ears)**	**χ^2^**	**P**
cVEMP	56	2	5.949	0.015	54	4	0.904	0.342
oVEMP	48	10			51	7		

### Self-comparison of cVEMP and oVEMP elicit rates in the RVC group

The cVEMP and oVEMP elicit rates of the RVC group are shown in Table 4. The elicit rate of ACS-cVEMP was 98%, which was similar to the 90% value of ACS-oVEMP, with no statistical significance in the comparison (χ^2^ = 1.914, *P* = 0.167). The GVS-cVEMP and GVS-oVEMP elicit rates of the RVC group were 98 and 95%, respectively, with no statistical significance in the comparison (χ^2^ = 0.346, *P* = 0.556).

**Table 4:** Self-comparison of cVEMP and oVEMP elicit rates in the RVC group.

**VEMPs**	**ACS**				**GVS**			
	**Elicite (ears)**	**Not elicite (ears)**	**χ^2^**	**P**	**Elicite (ears)**	**Not elicite (ears)**	**χ^2^**	**P**
cVEMP	41	1	1.914	0.167	41	1	0.346	0.556
oVEMP	38	4			40	2		

### Comparison of latencies and intervals of cVEMP

**Figure 3:**
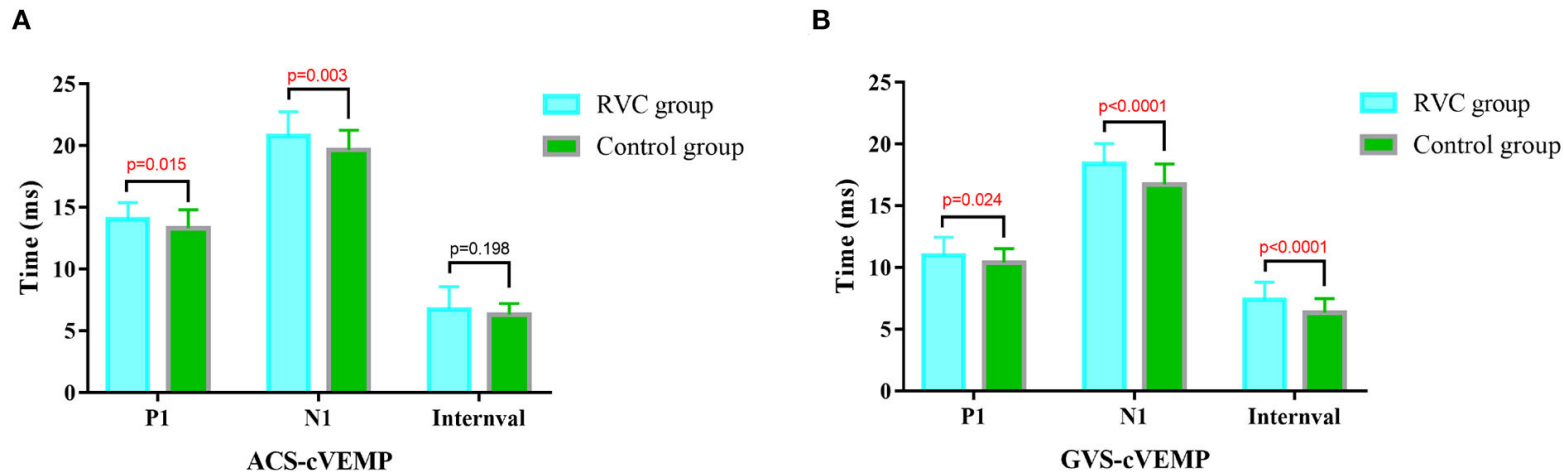
Comparison of latencies and intervals of cVEMPs between the RVC group and the Control group. **(A)** Comparison of typical ACS-cVEMP waveforms. **(B)** Comparison of typical GVS-cVEMP waveforms.

The cVEMP P1 latencies, N1 latencies, and intervals of the two groups are shown in Figure 3. The P1 and N1 latencies of ACS-cVEMP and GVS-cVEMP in the RVC group were longer than those in the control group, with statistical significance in the comparison (*P* < 0.05). The interval of GVS-cVEMP in the RVC group was longer than that in the control group, with statistical significance in the comparison (*P* < 0.05). But the interval of ACS-cVEMP was similar between the two groups, with no statistical significance in the comparison (*P* > 0.05).

### Comparison of latencies and intervals of oVEMP

**Figure 4:**
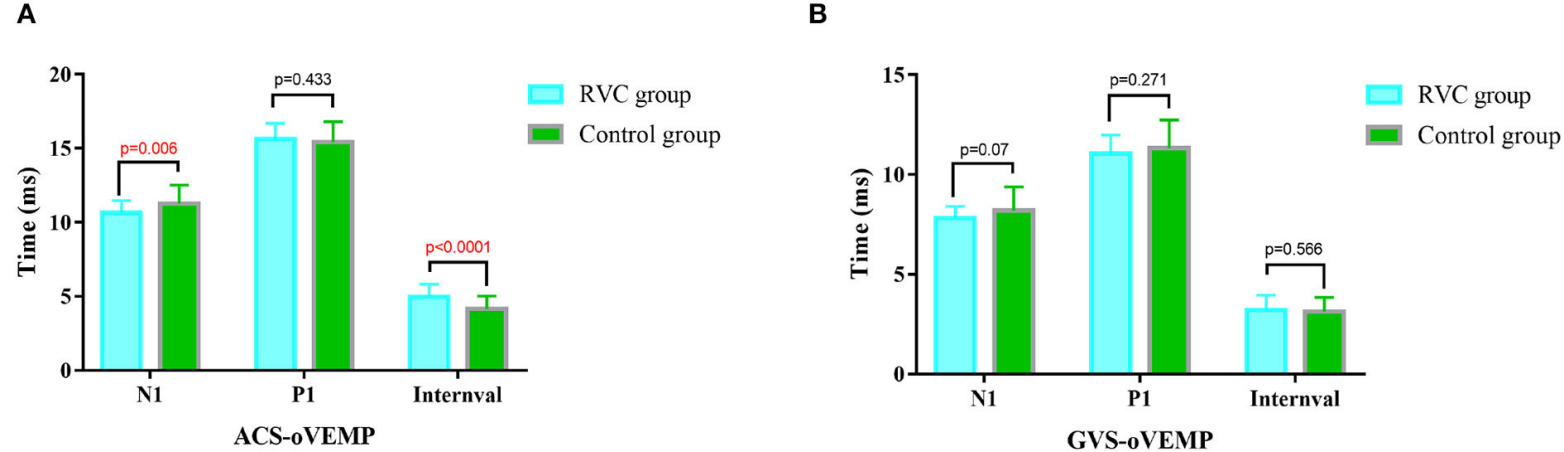
Comparison of latencies and intervals of oVEMPs between the RVC group and the Control group. **(A)** Comparison of typical ACS-oVEMP waveforms. **(B)** Comparison of typical GVS-oVEMP waveforms.

The oVEMP P1 latencies, N1 latencies, and intervals of the two groups are shown in Figure 4. The N1 latency of ACS-oVEMP in the RVC group was statistically shorter than that in the control group (*P* < 0.05), while the P1 latency was not statistically different from that in the control group (*P* > 0.05). The interval of ACS-oVEMP in the RVC group was statistically longer than that in the control group (*P* < 0.05). The N1 latency, P1 latency, and interval of GVS-oVEMP in the RVC group were not statistically different from those in the control group (*P* > 0.05).

### Comparison of amplitudes and AARs of cVEMP

The amplitudes and AARs of the two groups are shown in Table 5. The amplitude of ACS-cVEMP in the RVC group was significantly higher than that in the control group (*P* < 0.05). However, there was no significant difference in the AAR of ACS-cVEMP, the amplitude, and the AAR of GVS-cVEMP between the two groups (*P* > 0.05).

**Table 5:** Comparison of latencies and intervals of cVEMP between the RVC group and the control group.

**Group**	**ACS-cVEMP**		**GVS-cVEMP**	
	**Amplitude (μV)**	**AAR (%)**	**Amplitude (μV)**	**AAR (%)**
RVC	182.08 ± 71.21	13.04 ± 12.11	145.46 ± 54.41	23.07 ± 25.14
Control	102.83 ± 64.34	15.86 ± 13.14	129.19 ± 64.54	19.48 ± 15.99
T	−5.727	0.751	−1.3	−0.596
P	<0.0001	0.457	0.197	0.554

### Comparison of amplitudes and AAR values of oVEMP

The amplitudes and AARs of the two groups are shown in Table 6. The amplitude of ACS-oVEMP in the RVC group was not significantly different from that in the control group (*P* > 0.05). The AAR value of ACS-oVEMP in the RVC group was higher than that in the control group (*P* < 0.05). The amplitude of GVS-oVEMP in the RVC group was significantly higher than that in the control group (*P* < 0.05). The AAR value of GVS-oVEMP in the RVC group was not significantly different from that in the control group (*P* > 0.05).

**Table 6:** Comparison of latencies and intervals of oVEMP between the RVC group and the control group.

**Group**	**ACS-oVEMP**		**GVS-oVEMP**	
	**Amplitude (μV)**	**AAR (%)**	**Amplitude (μV)**	**AAR (%)**
RVC	4.94 ± 2.51	20.01 ± 10.74	7.32 ± 6.16	17.51 ± 17.09
Control	4.44 ± 3.30	11.67 ± 10.15	4.61 ± 2.36	18.49 ± 12.35
T	−0.776	−2.491	−2.631	−0.21
*P*	0.4405	0.017	0.011	0.834

## Discussion

Epidemiological studies have shown that RVC is the most common cause of dizziness or vertigo in children (15–17), and the etiology and pathogenesis of the disease are still not well understood and were even controversial.

Eviatar first found in a study of 24 children with vertigo as their chief complaint that vestibular damage could be a peripheral vestibular system lesion (18). However, Finkelhor concluded that the most likely etiology of RVC is a transient ischemic disturbance of the central vestibular system secondary to a vascular disturbance of the posterior circulation after summarizing the previous literature and analyzing five cases he encountered (19). Lanzi examined the clinical aspects of RVC in infancy and its most common differential diagnosis, particularly the analogies and differences with the later onset form of “migraine”, and concluded that RVC can be interpreted as a migraine precursor and MV as a migraine equivalent (20). Salami et al. investigated the diagnostic role of the visual vestibular interaction test for vertigo in children and suggested that the visual system of newborns is immature at birth and continues to develop until maturity in childhood and that this transient “abnormality” during development may lead to a failure of binocular information pooling and thus to vertigo in children (21). The latest review on “Prevalence and diagnosis of vestibular disorders in children” concluded that most of the current theories on the pathogenesis of RVC are still based on clinical studies assessing the vestibular system (22).

VEMP is often used to evaluate the function of the saccule and the integrity of the saccule–colic reflex (SCR) pathway (23). oVEMP is often applied to evaluate the function of the utricle and the integrity of the vestibulo-ocular reflex (VOR) pathway (23). ACS-VEMPs depend on the integrity of the otolith, while GVS directly stimulates the vestibular nerve endings to elicit VEMPs (24, 25). Therefore, different VEMPs can be compared to locate intra-labyrinthine and retro-labyrinthine lesions (26). If GVS-VEMPs can be elicited and ACS-VEMPs cannot be elicited, then the lesion is located in the otolith. If both ACS-VEMPs and GVS-VEMPs cannot be elicited, it is likely to be a retro-labyrinthine lesion.

The results of our study showed that there was no statistical difference in the elicit rates of cVEMP and oVEMP under ACS and GVS stimulation in the RVC group, suggesting that the function of the peripheral otolithic end receptors and their pathways is complete in children with RVC. The P1 and N1 latencies of ACS-cVEMP and GVS-cVEMP in children with RVC were longer than those in the control group, while the N1 latency of ACS-oVEMP was shorter than that in the control group, and no prolongation was seen in the P1 latency of ACS-oVEMP or in the latencies of GVS-oVEMP. Among them, P13 shortening did not have much clinical significance but was more of a statistical difference. The prolongation of P13 has clinical significance, suggesting that there might be impairment in the saccule and the inferior vestibular nerve conduction pathway in children with RVC, while the utricle and the superior vestibular nerve conduction pathway are not affected. Lin et al. (6) found that among 15 children with RVC, 73% had prolonged ACS-cVEMP latencies, which was significantly different from healthy children, while ACS-oVEMPs were all elicited normally and did not differ from healthy children. Therefore, they hypothesized that the VOR pathway and upper brainstem were functioning normally, while the vestibulospinal reflexes of the saccule–inferior vestibular pathway may have abnormal lesions. Chang et al. (5) performed the caloric test and cVEMP tests in children with RVC and normal children and found that the rate of abnormal cVEMP was significantly higher in children with RVC than that in the caloric test, which led to the assumption that there might be some lesions in the inferior vestibular conduction pathway in children with RVC. The caloric test detects the response of the horizontal semicircular canal to low-frequency stimuli and assess the superior vestibular conduction pathway. Although previous studies have proposed a mechanism of damage in the inferior vestibular conduction pathway in patients with RVC, they have not been able to define whether this abnormality originates from the saccule or in retro-labyrinthine. Our study further investigated the possibility of intra-labyrinthine or retro-labyrinthine vestibular damage in children with RVC based on ACS-VEMPs and GVS-VEMPs. Combined with these findings, we speculated that the retro-labyrinthine portion and lower brainstem along the SCR pathway were impaired in children with RVC.

Murofushi et al. (27, 28) proposed a “neuritis pattern” as a theoretical mechanism for retro-labyrinthine injury of the inferior vestibular nerve conduction pathway, including the inferior vestibular nerve, lateral vestibular nucleus, medial vestibulospinal tract, paracentral nucleus, and paracentral nerve. In addition, in a study of investigating the diagnostic value of vestibular test and the high stimulus rate auditory brainstem response (ABR) test and the possible mechanism responsible for RVC, Zhang et al. (7) proposed that the vascular mechanism might be involved in the pathogenesis of RVC, that is, the ischemia of vestibular nuclei and vestibular pathway was one of the causes, and the inferior vestibular nerve pathway was more vulnerable than the superior vestibular nerve pathway. Batuecas-Caletrío et al. (29) observed a higher prevalence of migraine in patients with RVC than in the general population and suggested that RVC is a precursor to migraine in childhood. Marcelli et al. (30) further reported their 10-year follow-up study of 17 children with RVC, with 10 of them with migraine.

However, as reviewed by Lempert et al. (31), genetic, neurochemical, and inflammatory mechanisms may be involved in the pathophysiological mechanisms of VM. The patients' genetic susceptibility leads to a more excitable and vulnerable cerebral cortex, which produces a local neurogenic inflammatory response when relevant triggers are present in the environment, resulting in increased sensitivity of peripheral and central afferent nerve conduction pathways, thereby activating migraine-related loops and the trigeminal innervated vascular system (32). Most neurotransmitters involved in the pathogenesis of migraine, such as calcitonin gene-related peptides, 5-hydroxytryptamine, norepinephrine, and dopamine, also modulate the activity of central and peripheral vestibular neurons and may be involved in the pathogenesis of VM (31). Aseptic inflammation of intracranial vessels, such as lesions of the vascular striatum trigeminal, cochlear spiral cochlear axial artery, and dark cell area of the jugular crest, causes inner ear damage, which leads to the appearance of vertigo (31, 32). Therefore, more in-depth research is needed to explore the pathogenesis of RVC and its correlation with VM in the follow-up. We will also conduct a systematic follow-up study of this group of children with RVC in this study to further investigate the prognosis of the disease and the changes of the parameters of VEMPs.

Several studies have shown (33, 34) that the amplitude of VEMPs fluctuates greatly, which is related to the subjects' muscle tone. To avoid the influence of muscle tone on the results, we further compared the AARs of the subjects' binaural VEMPs in our study. Statistical analysis revealed that the AAR values of ACS-cVEMPs and GVS-VEMPs in the RVC group were similar to those in the control group. The AAR value of ACS-oVEMP in the RVC group was significantly higher than that in the control group. But the mean AAR value in the RVC group was still within the normal range, suggesting that the function of the bilateral utricle and superior vestibular nerve conduction pathway in children with RVC was affected to some extent but not impaired. At the same time, the reasons of poor cooperation in the oVEMP test, the relatively insensitivity of young children to stimulation sounds, testing errors, and so on cannot be ruled out.

## Conclusion

The elicit rates of VEMPs in children with RVC are the same as those in healthy children, with no significant reduction in amplitude, and the bilateral AAR is still within the normal range. Both ACS-cVEMP and GVS-cVEMP latencies were significantly prolonged in children with RVC; however, the elicit rate is no different from that in the control group, suggesting that there might be potential impairment in the inferior vestibular nerve and the subsequent nerve conduction pathway in them without affecting the utricle and the superior vestibular nerve conduction pathway.

## Data availability statement

The raw data supporting the conclusions of this article will be made available by the authors, without undue reservation.

## Ethics statement

The studies involving human participants were reviewed and approved by the Ethical Committee of the Xinhua Hospital. Written informed consent to participate in this study was provided by the participants' legal guardian/next of kin.

## Author contributions

JC and JY contributed to the study design. JS and LW preformed VEMP tests. XS and DG contributed to statistical analysis and manuscript draft. All authors helped to perform the analysis and to revise the manuscript with constructive discussions. All authors listed have contributed sufficiently to the project to be included as authors, and all those who are qualified to be authors are listed in the author byline.

## Funding

This work was supported by Science and Technology Commission Foundation of Shanghai (No. 21Y31900504), and the Hospital Funded Clinical Research, Xin Hua Hospital Affiliated to Shanghai JiaoTong University School of Medicine, Clinical Research Unit (No. 21XHDB02, 2021).

## Conflict of interest

The authors declare that the research was conducted in the absence of any commercial or financial relationships that could be construed as a potential conflict of interest.

## Publisher's note

All claims expressed in this article are solely those of the authors and do not necessarily represent those of their affiliated organizations, or those of the publisher, the editors and the reviewers. Any product that may be evaluated in this article, or claim that may be made by its manufacturer, is not guaranteed or endorsed by the publisher.
